# A non-interventional cross-sectional re-contact study investigating the relationship between overactive bladder and frailty in older adults in Japan

**DOI:** 10.1186/s12877-022-02756-7

**Published:** 2022-01-21

**Authors:** Masaki Yoshida, Shosuke Satake, Kota Ishida, Yusuke Tanaka, Masashi Ukai

**Affiliations:** 1 Department of Urology, National Center for Geriatrics and Gerontology, Obu, Aichi Japan; 2 Department of Geriatric Medicine, National Center for Geriatrics and Gerontology, Obu, Aichi Japan; 3 Medical Affairs, Astellas Pharma Inc., Tokyo, Japan; 4 Data Science, Development, Astellas Pharma Inc., Tokyo, Japan

**Keywords:** Overactive bladder, Frailty, Older adults, Japan

## Abstract

**Background:**

Increasing age is associated with frailty and a higher prevalence of overactive bladder (OAB). Given the rapidly increasing proportion of older adults in Japan, a better understanding of the relationship between frailty and OAB is needed to inform future healthcare planning. This study assessed the association between frailty and OAB in older adults in Japan and evaluated the impact on their health-related quality of life (HRQoL).

**Methods:**

This was a cross-sectional re-contact study of respondents who previously completed the National Health and Wellness Survey 2018 in Japan. Participants were aged ≥65 years and Japanese speakers and readers. As part of a customized online survey, participants were screened for frailty using the Kihon Checklist (frail = scores ≥8 points) and OAB using the overactive bladder symptom score (OAB = total score ≥ 3 points and ≥ 2 points on question 3). The primary endpoint was the odds ratio of frailty in older adults with and without OAB assessed using a multivariable logistic regression model. Secondary endpoints were the prevalence rates of OAB and frailty. Exploratory endpoints assessed HRQoL using the Medical Outcomes Study 12-Item Short Form Survey Instrument version 2 (SF-12v2).

**Results:**

Overall, 2953 participants were included: 150 (5.1%) were frail OAB, 416 (14.1%) non-frail OAB, 287 (9.7%) frail non-OAB, and 2100 (71.1%) non-frail non-OAB. There was a statistically significant correlation between frailty and OAB demonstrated by an adjusted odds ratio (95% CI) of 2.78 (2.18–3.54; *p* <  0.001). The prevalence (95% CI) of OAB was 34.3% (29.9–38.8) in frail and 16.5% (15.1–18.0) in non-frail older adults; the prevalence of frailty was 26.5% (22.9–30.1) and 12.0% (10.7–13.3) in older adults with and without OAB. HRQoL was assessed in 150 participants per group. The adjusted HRQoL analyses showed significantly lower scores in participants who were frail OAB vs. frail non-OAB for most of the SF-12v2 scores/sub-component scores.

**Conclusions:**

These data highlight the statistically significant positive correlation between frailty and OAB among older adults in Japan and may provide valuable information on the burden of OAB and frailty on older adults to healthcare professionals when considering future healthcare planning.

**Supplementary Information:**

The online version contains supplementary material available at 10.1186/s12877-022-02756-7.

## Background

Worldwide, Japan has the highest percentage of its population that is at least 65 years of age (28.7%) [1]. The majority of this age group are women and there are ~ 80,000 individuals who are at least 100 years old. Residents of Japan have a high life expectancy and the government is acting to address the consequences of an aging population, including adequate healthcare provision [1].

Increasing age is associated with increasing frailty, defined as a multifactorial geriatric syndrome characterized by age-associated reductions in physiologic reserve and function across multi-organ systems [2]. The prevalence of frailty in Japan in those aged 65 years or older has been estimated at between 7.4 and 21.6% [3, 4] and frailty is associated with a high risk of morbidity and mortality [2].

Overactive bladder (OAB) syndrome is defined as “urinary urgency, usually accompanied by increased daytime frequency and/or nocturia, with urinary incontinence (OAB-wet) or without (OAB-dry), in the absence of urinary tract infection or other detectable disease” [5]. The prevalence of OAB in Japan is ~ 12.4% among adults aged ≥40 years and increases with age from 5% in those aged 40–49 to 37% in those aged 80 years or over [6, 7]. OAB can place a significant burden on individuals, in terms of adversely affecting their quality of life, mood (anxiety and depression), and daily activities and also has an impact on healthcare costs [8–11].

Although an association between frailty and OAB has been demonstrated in non-Japanese populations [12], a better understanding of the relationship is needed to inform future healthcare planning given the rapidly increasing proportion of older adults in the Japanese population. Hence, the objectives of this study were to assess the association between frailty and OAB in older adults in Japan and to evaluate the impact on their health-related quality of life (HRQoL).

## Methods

### Study design and endpoints

This was a cross-sectional re-contact study of respondents who had previously completed the National Health and Wellness Survey (NHWS) 2018 in Japan [13]. The NHWS is a cross-sectional survey conducted worldwide (Japan, USA, France, Germany, Italy, Spain, UK, China, South Korea, Taiwan, Russia, and Brazil). In Japan, the NHWS is a self-administered, internet-based questionnaire that is completed by respondents aged at least 18 years who are members of the Kantar Profiles global panel or its panel partners, which are general purpose, web-based opt-in consumer panels. However, the demographic data provided can be used for healthcare purposes [13]. A stratified random sampling procedure was implemented to ensure that the demographic composition of the NHWS sample was representative of the adult population in Japan in terms of sex and age. Approval for this study was provided by a Central IRB (Pearl IRB, Indianapolis, IN, USA).

The primary endpoint of the study was the odds ratio of frailty in older adults with OAB to that of frailty in older adults without OAB. Secondary endpoints were the prevalence of older adults with OAB in the frail vs. non-frail groups, and the prevalence of older adults who are frail with OAB vs. without OAB. Exploratory endpoints were the Medical Outcomes Study 12-Item Short Form Survey Instrument version 2 (SF-12v2™, standard version) [14] Japanese norm-based Physical Component Summary (PCS), Mental Component Summary (MCS), Role Component Summary (RCS), and 6-dimensional health state classification (SF-6D) score, and scores for the eight SF-12v2 health domains.

### Participants and assessments

Participants were included if they were 65 years or older, speak and read Japanese, and provided informed consent to participate. Participants who required long-term care or support from the Japanese Ministry of Health, Labor and Welfare (MHLW) were excluded. Participants could withdraw from the study for any reason, at any time, without giving a reason for doing so and without penalty or prejudice, and anyone who withdrew consent for their data to be used in the study was discontinued.

**Fig. 1:**
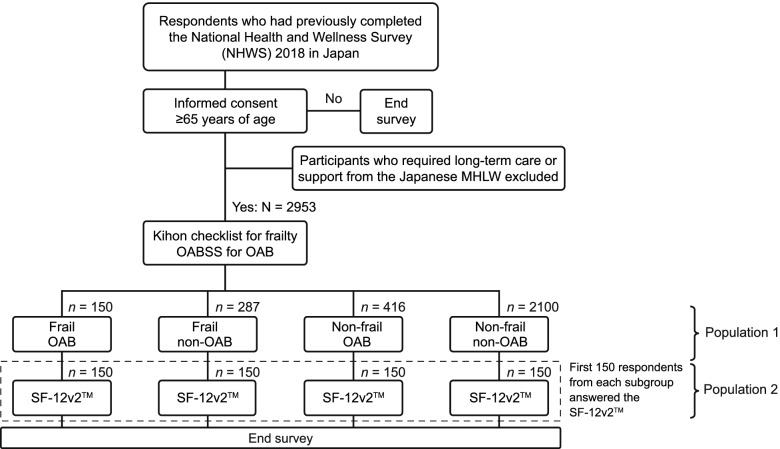
Study design and patient disposition. MHLW, Ministry of Health, Labor and Welfare; OAB, overactive bladder; OABSS, overactive bladder symptom score; SF-12v2, Medical Outcomes Study 12-Item Short Form Survey Instrument version 2

As part of a customized online survey, participants were screened for frailty using the Kihon Checklist [15] and for OAB using the overactive bladder symptom score (OABSS) [16] (Fig. 1). The Kihon Checklist was developed by the Japanese MHLW to assess frailty in older adults and initiate appropriate care needs. There are 25 questions regarding physical strength, nutrition, eating, socialization, memory, mood, and lifestyle which are answered yes/no and scored 0 (pass) or 1 (fail). The maximum score of 25 indicates severe frailty, and in this study frailty was defined as a score of ≥8 points [15]. The OABSS is a tool for assessing OAB symptoms. The sum score is obtained from the responses to four questions regarding the frequency of daytime voiding, nighttime voiding, urgency, and urgency incontinence, with a maximum score of 15 [16]. Participants were classified as having OAB if they had a total of ≥3 points on the OABSS and ≥ 2 points on question 3 of the OABSS. Four subgroups were established based on the responses: frail OAB, non-frail OAB, frail non-OAB, and non-frail non-OAB. These participants comprised population 1. Data collection was completed when each of the four subgroups reached at least the target sample size (*N* = 150). The target sample size was determined to provide 80% power to detect an effect size of 0.325 for HRQoL scores between frail OAB and frail non-OAB using a two-tailed *t*-test at the 5% significance level. Conventionally, an effect size of 0.2 is considered small, 0.5 is considered medium, and 0.8 is considered large. Hence this target sample size was able to detect a small to medium effect size.

The SF-12v2 is a validated scale for assessing HRQoL. This questionnaire uses eight health domains summarized into three scores (PCS, MCS, and RCS) for the Japanese population using the Japanese norm-based scoring. The SF-12v2 is also used to generate health state utilities by applying the SF-6D algorithm [14, 17–19]. Participants were invited to complete the SF-12v2 [14] on a sequential basis until HRQoL data had been collected from the target sample size of 150 for each subgroup (Fig. 1). These participants comprised population 2.

Sociodemographic (age, sex, education, annual household income, living status), clinical characteristics (Charlson comorbidity index [non-age adjusted version [20]]), and lifestyle information (smoking status, alcohol use, exercise behavior) were extracted from the 2018 NHWS Japan database and combined with data from the customized survey by matching the respondents’ panel IDs.

### Statistical analyses

For the demographic and clinical characteristics, continuous variables were summarized as the mean and standard deviation (SD) and categorical variables as the frequency (percentage).

For the primary endpoint, a crude odds ratio was calculated and the Chi-square test applied at the level of significance of 0.05. A multivariable logistic regression model with frailty status as the dependent variable and OAB status as the independent variable was then used to generate odds ratios (95% CI) for the relationship between OAB and frailty. Backward elimination was used to determine which variables remained in the final model (*p* <  0.15 to retain), while prespecified variables of clinical importance (age, sex, living status, Charlson comorbidity index, exercise behavior) were retained regardless of the *p*-value. The prespecified variables of clinical importance were determined following discussions with medical experts in the fields of urology and geriatrics and were based on their latest knowledge and experiences.

The prevalence of OAB in frail and non-frail older adults was estimated as the number who had OAB divided by the number in the frail and non-frail groups, respectively. Similarly, the prevalence of frailty in older adults with OAB and without OAB was estimated as the number who were frail divided by the number who had OAB and who did not have OAB, respectively.

For the exploratory endpoint (HRQoL), mean and SD were used to summarize the data. One-way ANOVA weighted with a sampling ratio from population 1 to population 2 was used to assess differences in HRQoL. In addition, among frail older participants, generalized linear models with normal distributions and identity link function and weighted with the sampling ratio were used, with HRQoL as the dependent variable and OAB status as the independent variable. Like the method used for the primary analysis, the variables included in the final model were selected using backward elimination, with SF-6D as the dependent variable.

An additional analysis was undertaken to assess the association between frailty status and OAB severity. For frailty, robust was defined as a Kihon Checklist total score of 0–3 and pre-frail as a total score of 4–7. Severe/moderate OAB was defined as a total OABSS of ≥6 and mild OAB as a total OABSS ≤5.

## Results

A total of 2953 participants gave consent to be included in the study. Of these, 437 were classed as frail and 2516 as non-frail, while 566 had OAB and 2387 were non-OAB; the numbers in each subgroup were frail OAB, *n* = 150; non-frail OAB, *n* = 416; frail non-OAB, *n* = 287; and non-frail non-OAB, *n* = 2100 (population 1). As per the study design, there were 150 participants in each of the four subgroups in population 2 (Fig. 1). The demographic and clinical characteristics of the overall populations and subgroups are shown in Table 1. In the overall group the majority were male (65.0%), were married/living with a partner (81.4%), and had a university degree (58.6%). Further details of OABSS scores and severity are given in Supplementary Table 1, Additional File 1.

**Table 1:** Demographic characteristics

**Population 1**	**Total (*N*** **= 2953)**	**OAB (*n*** **= 566)**		**Non-OAB (*n*** **= 2387)**	
		**Frail (*n*** **= 150)**	**Non-frail (*n*** **= 416)**	**Frail (*n*** **= 287)**	**Non-frail (*n*** **= 2100)**
Mean (SD) age, years	71.7 (4.2)	71.5 (4.6)	72.7 (4.4)	71.4 (4.1)	71.6 (4.1)
**Sex**					
Male	1919 (65.0)	110 (73.3)	330 (79.3)	192 (66.9)	1287 (61.3)
Female	1034 (35.0)	40 (26.7)	86 (20.7)	95 (33.1)	813 (38.7)
**Relationship status**					
Married/living with partner	2403 (81.4)	111 (74.0)	363 (87.3)	228 (79.4)	1701 (81.0)
Single/divorced/separated/widowed/ declined to answer	550 (18.6)	39 (26.0)	53 (12.7)	59 (20.6)	399 (19.0)
**Education**					
University degree	1729 (58.6)	77 (51.3)	287 (69.0)	142 (49.5)	1223 (58.2)
No/declined to answer	1224 (41.4)	73 (48.7)	129 (31.0)	145 (50.5)	877 (41.8)
**Household income**					
< ¥3,000,000	642 (21.7)	40 (26.7)	77 (18.5)	83 (28.9)	442 (21.0)
¥3,000,000 to < ¥5,000,000	982 (33.3)	50 (33.3)	150 (36.1)	89 (31.0)	693 (33.0)
¥5,000,000 to < ¥8,000,000	603 (20.4)	29 (19.3)	98 (23.6)	42 (14.6)	434 (20.7)
¥8,000,000 or more	342 (11.6)	8 (5.3)	58 (13.9)	29 (10.1)	247 (11.8)
Declined to answer	384 (13.0)	23 (15.3)	33 (7.9)	44 (15.3)	284 (13.5)
**Living status**					
Live alone (NHWS)	386 (13.1)	24 (16.0)	35 (8.4)	38 (13.2)	289 (13.8)
Live with someone (NHWS)	2567 (86.9)	126 (84.0)	381 (91.6)	249 (86.8)	1811 (86.2)
**Mean (SD) Charlson comorbidity index**	0.28 (0.60)	0.47 (0.78)	0.31 (0.62)	0.40 (0.74)	0.24 (0.55)
**BMI**					
< 18.5	214 (7.2)	11 (7.3)	18 (4.3)	33 (11.5)	152 (7.2)
≥ 18.5–< 23	1497 (50.7)	54 (36.0)	203 (48.8)	117 (40.8)	1123 (53.5)
≥ 23–< 25	707 (23.9)	36 (24.0)	115 (27.6)	59 (20.6)	497 (23.7)
> 25	535 (18.1)	49 (32.7)	80 (19.2)	78 (27.2)	328 (15.6)
**Parkinson’s disease**					
Not experienced	2951 (99.9)	149 (99.3)	416 (100)	287 (100)	2099 (100)
Experienced	2 (0.1)	1 (0.7)	0	0	1 (0.0)
**Falls**					
No fall in the last year	2181 (73.9)	80 (53.3)	302 (72.6)	154 (53.7)	1645 (78.3)
Fall in the last year	772 (26.1)	70 (46.7)	114 (27.4)	133 (46.3)	455 (21.7)
**Smoking status**					
Never	1400 (47.4)	55 (36.7)	167 (40.1)	118 (41.1)	1060 (50.5)
Former	1068 (36.2)	63 (42.0)	189 (45.4)	101 (35.2)	715 (34.0)
Current	485 (16.4)	32 (21.3)	60 (14.4)	68 (23.7)	325 (15.5)
**Use of alcohol**					
Abstain	861 (29.2)	46 (30.7)	105 (25.2)	99 (34.5)	611 (29.1)
Currently consume	2092 (70.8)	104 (69.3)	311 (74.8)	188 (65.5)	1489 (70.9)
**Vigorous exercise in past 30 days**					
No	1335 (45.2)	91 (60.7)	161 (38.7)	179 (62.4)	904 (43.0)
Yes	1618 (54.8)	59 (39.3)	255 (61.3)	108 (37.6)	1196 (57.0)
**Population 2**	**Total (*n*** **= 600)**	**OAB (*n*** **= 300)**		**Non-OAB (*n*** **= 300)**	
		**Frail (*n*** **= 150)**	**Non-frail (*n*** **= 150)**	**Frail (*n*** **= 150)**	**Non-frail (*n*** **= 150)**
**Mean (SD) age, years**	71.5 (4.4)	71.5 (4.6)	72.2 (4.5)	71.3 (4.4)	71.0 (4.1)
**Sex**					
Male	481 (80.2)	110 (73.3)	138 (92.0)	119 (79.3)	114 (76.0)
Female	119 (19.8)	40 (26.7)	12 (8.0)	31 (20.7)	36 (24.0)
**Relationship status**					
Married/living with partner	479 (79.8)	111 (74.0)	126 (84.0)	121 (80.7)	121 (80.7)
Single/divorced/separated/widowed/ declined to answer	121 (20.2)	39 (26.0)	24 (16.0)	29 (19.3)	29 (19.3)
**Level of education**					
University degree	368 (61.3)	77 (51.3)	111 (74.0)	79 (52.7)	101 (67.3)
No/declined to answer	232 (38.7)	73 (48.7)	39 (26.0)	71 (47.3)	49 (32.7)
**Household income**					
< ¥3,000,000	141 (23.5)	40 (26.7)	27 (18.0)	44 (29.3)	30 (20.0)
¥3,000,000 to < ¥5,000,000	205 (34.2)	50 (33.3)	49 (32.7)	50 (33.3)	56 (37.3)
¥5,000,000 to < ¥8,000,000	120 (20.0)	29 (19.3)	38 (25.3)	23 (15.3)	30 (20.0)
¥8,000,000 or more	72 (12.0)	8 (5.3)	25 (16.7)	17 (11.3)	22 (14.7)
Declined to answer	62 (10.3)	23 (15.3)	11 (7.3)	16 (10.7)	12 (8.0)
**Living status**					
Live alone (NHWS)	82 (13.7)	28 (18.7)	18 (12.0)	19 (12.7)	22 (14.7)
Live with someone (NHWS)	518 (86.3)	122 (81.3)	132 (88.0)	131 (87.3)	128 (85.3)
**Mean (SD) Charlson comorbidity index**	0.39 (0.72)	0.47 (0.78)	0.35 (0.69)	0.47 (0.81)	0.26 (0.56)
**BMI**					
< 18.5	39 (6.5)	11 (7.3)	6 (4.0)	11 (7.3)	11 (7.3)
≥18.5–< 23	277 (46.2)	54 (36.0)	71 (47.3)	61 (40.7)	91 (60.7)
≥23–< 25	137 (22.8)	36 (24.0)	40 (26.7)	30 (20.0)	31 (20.7)
> 25	147 (24.5)	49 (32.7)	33 (22.0)	48 (32.0)	17 (11.3)
**Parkinson’s disease**					
Not experienced	599 (99.8)	149 (99.3)	150 (100)	150 (100)	150 (100)
Experienced	1 (0.2)	1 (0.7)	0	0	0
**Falls**					
No fall in the last year	387 (64.5)	80 (53.3)	104 (69.3)	83 (55.3)	120 (80.0)
Fall in the last year	213 (35.5)	70 (46.7)	46 (30.7)	67 (44.7)	30 (20.0)
**Smoking status**					
Never	223 (37.2)	55 (36.7)	54 (36.0)	53 (35.3)	61 (40.7)
Former	271 (45.2)	63 (42.0)	78 (52.0)	60 (40.0)	70 (46.7)
Current	106 (17.7)	32 (21.3)	18 (12.0)	37 (24.7)	19 (12.7)
**Use of alcohol**					
Abstain	147 (24.5)	46 (30.7)	27 (18.0)	42 (28.0)	32 (21.3)
Currently consume	453 (75.5)	104 (69.3)	123 (82.0)	108 (72.0)	118 (78.7)
**Vigorous exercise in the past 30 days**					
No	284 (47.3)	91 (60.7)	45 (30.0)	92 (61.3)	56 (37.3)
Yes	316 (52.7)	59 (39.3)	105 (70.0)	58 (38.7)	94 (62.7)

*Abbreviations: BMI* Body mass index, *NHWS* National Health and Wellness Survey

n (%) unless noted otherwise; living status in population 2 was calculated based on the customized online survey

Analysis of the primary endpoint found a statistically significant correlation between frailty status and OAB status in this population of older adults (*p* <  0.001); 5.1% were frail OAB, 9.7% were frail non-OAB, 14.1% were non-frail OAB, and 71.1% were non-frail non-OAB. The crude odds ratio (95% CI) was 2.64 (2.11–3.30). Using logistic regression (frailty status dependent variable; OAB status independent variable) and adjusting for age, sex, level of education, household income, living status, Charlson comorbidity index, body mass index, smoking status, use of alcohol, and exercise behavior, the odds ratio was 2.78 (2.18–3.54; *p* <  0.001; Table 2).

**Table 2:** Logistic regression for the odds ratio between OAB and frailty status, with frailty status as dependent variable

Parameter	Odds ratio	95% CI	***p***-Value
**OAB status**			
No	Reference		< 0.001
Yes	2.776	2.179–3.538	
**Age per 1 unit**	0.981	0.955–1.008	0.161
**Charlson comorbidity index per 1 unit**	1.391	1.191–1.626	< 0.001
**Sex**			
Male	Reference		0.750
Female	0.954	0.715–1.273	
**Level of education**			
University degree	Reference		0.001
No/declined to answer	1.480	1.183–1.852	
**Household income**			
< ¥3,000,000	Reference		0.005
¥3,000,000 to < ¥5,000,000	0.739	0.547–0.999	
¥5,000,000 to < ¥8,000,000	0.593	0.416–0.845	
¥8,000,000 or more	0.530	0.342–0.822	
Declined to answer	0.981	0.683–1.411	
**Living status (NHWS)**			
Live alone	Reference		0.402
Live with someone	1.159	0.821–1.636	
**BMI category (Kihon checklist)**			
< 18.5	Reference		< 0.001
≥ 18.5–< 23	0.489	0.329–0.724	
≥ 23–< 25	0.559	0.363–0.860	
≥ 25	1.044	0.684–1.594	
**Smoking status**			
Never	Reference		0.012
Former	1.148	0.868–1.518	
Current	1.604	1.169–2.203	
**Use of alcohol**			
Abstain	Reference		0.118
Currently consume alcohol	0.822	0.642–1.051	
**Vigorous exercise in the past 30 days**			
No	Reference		< 0.001
Yes	0.506	0.405–0.633	

*Abbreviations: BMI* Body mass index, *NHWS* National Health and Wellness Survey, *OAB* overactive bladder

**Fig. 2:**
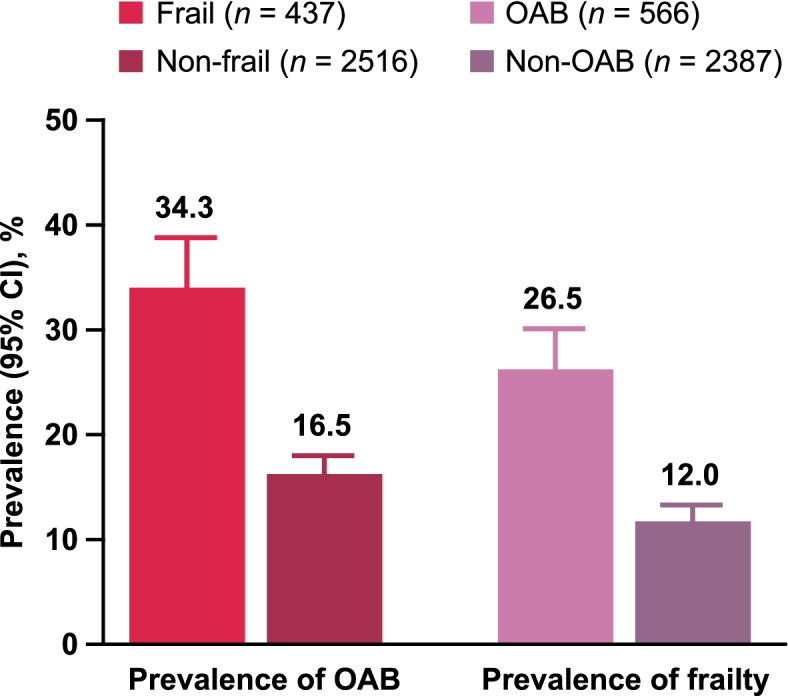
Prevalence of OAB and frailty by group. OAB, overactive bladder

**Fig. 3:**
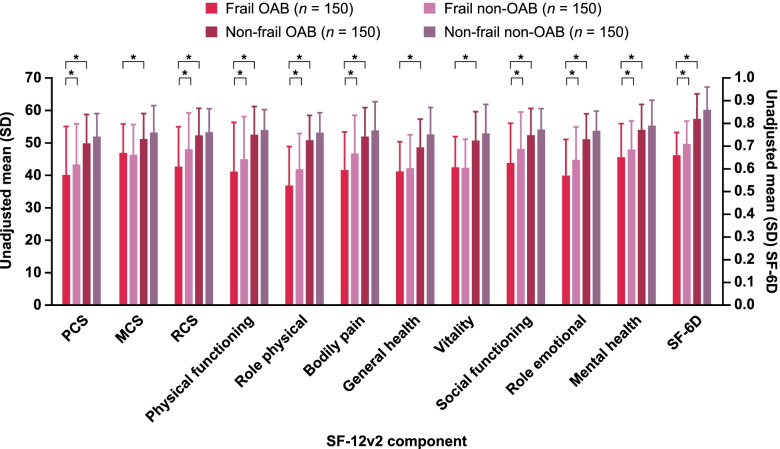
Unadjusted means of HRQoL for the OAB and frailty subgroups. HRQoL, health-related quality of life; MCS, Mental Component Summary; OAB, overactive bladder; PCS, Physical Component Summary; RCS, Role Component Summary; SF-12v2, Medical Outcomes Study 12-Item Short Form Survey Instrument version 2; SF-6D, 6-dimensional health state classification. *p < 0.05

**Fig. 4:**
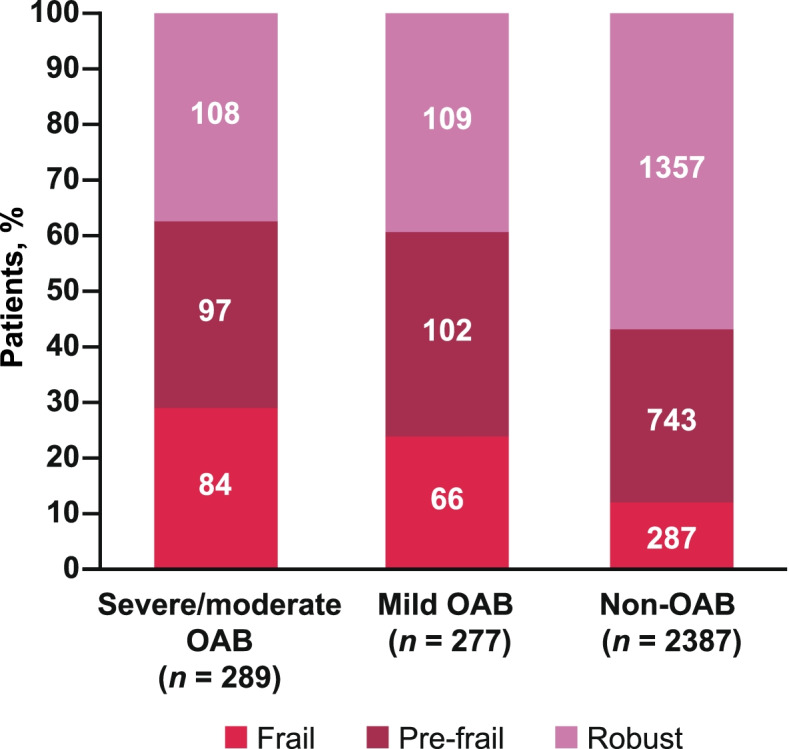
Relationship between OAB severity and frailty status. OAB, overactive bladder

The overall prevalence (95% CI) of OAB was 19.2% (17.7–20.6) and the prevalence of frailty was 14.8% (13.5–16.1). The prevalence estimates by group are shown in Fig. 2. The unadjusted analyses of HRQoL data in population 2 showed significantly lower scores in participants who were frail OAB compared with non-frail OAB across all scores and all sub-component scores (Fig. 3). Analyses adjusted for age, sex, household income, living status, Charlson comorbidity index, body mass index, and exercise behavior also showed that those who were frail OAB had significantly lower HRQoL scores than those who were frail non-OAB, with the exceptions of MCS, general health, and vitality (Supplementary Fig. 1, Additional File 2). Analysis of an association between frailty status and OAB severity suggested that the prevalence of frailty tends to increase in accordance with the severity of OAB (Fig. 4).

## Discussion

This study demonstrated a statistically significant correlation between frailty and OAB in older adults in Japan; logistic regression suggested that older adults with OAB were 2.78 times more likely to be frail than those without OAB. The prevalence of OAB was also higher among the frail (34.3%) compared with the non-frail group (16.5%). Similarly, the prevalence of frailty was higher among older adults with OAB (26.5%) compared with those without OAB (12.0%). Finally, there were significant differences in HRQoL between older adults with OAB who were frail and non-frail, and between older adults who were frail with and without OAB.

Pelvic floor weakness is one of the factors associated with OAB [21, 22]. Therefore, OAB may have a shared pathophysiology with frailty (especially, physical frailty). Previous studies have investigated an association between frailty and OAB, although the Timed Up and Go Test (TUGT) was used instead of the Kihon Checklist. The TUGT measures the time taken to get up from a chair, walk 3 m, turn, return, and sit down again. While it cannot assess all aspects of frailty, it has been shown to distinguish between frail and non−/pre-frail populations [23]. The method used to assess OAB also differed between studies; we used the OABSS, while other measures were the International Consultation on Incontinence female lower urinary tract symptoms questionnaire [24], medication dispensation records or self-reporting [25], and use of ICD codes [12, 25]. A prospective study in the United States found that individuals with OAB had slower TUGT results than those without OAB, and 32.3% of individuals with OAB were categorized as slow/frail compared with 11.0% without OAB [12]. A second prospective study of women receiving home care in Canada also reported a significant correlation between TUGT score and OAB at baseline and 3 months, with TUGT score also indicating fall risk [24]. Although not specifically categorized as being about frailty, a systematic review of falls and fractures associated with OAB found that 11.3 to 56.0% of patients with OAB experienced recurrent or serious falls and, when comparison with a non-OAB cohort was possible, there was a 1.3- to 2.3-fold increase in the risk of falls for OAB vs. non-OAB [25].

Our results are therefore consistent with those of others and provide evidence of an association between frailty and OAB. Although the TUGT was used in other studies assessing frailty, a systematic review confirmed that the Kihon Checklist is a reliable tool for predicting frailty. Furthermore, it is short and therefore easy to administer, assesses daily routine regardless of culture or living place, and is appropriate for use in cross-cultural studies [15, 26]. Similarly, the OABSS is a valid tool for describing OAB symptoms [16].

It is estimated that by 2060 40% of the Japanese population will be aged ≥65 years [27]. As frailty is considered to be a pre-disability state and potentially reversible, it is a target for both prevention and intervention to improve overall health outcomes [3]. Exercise is a key intervention for frailty [2] and may also help with symptoms of OAB through weight control [28]. In addition, a number of pharmacological treatments are available for OAB, but data are limited on the treatment of OAB among the frail older population. Reasons for this include exclusion from clinical trials, the vulnerability of this population, co-morbidities, and other medications [29–31]. A better understanding of the pathophysiology of OAB as well as the relationship between OAB and frailty will help to optimize treatment strategies for this population. OAB should potentially be considered as part of a geriatric syndrome, and many factors should be considered when determining how to manage OAB in the context of frailty to achieve the best outcome for an individual [29–31]. Given the anticipated rise in the older population in both Japan and worldwide [29], there will likely be an associated increase in the number of patients with OAB, with and without frailty. Further investigation of OAB and its management in the frail older population, including pharmacotherapy, is warranted, therefore [29, 30, 32, 33]. Recently, a treatment algorithm and guidelines for the management of older frail individuals with urinary incontinence have been developed by the 6th International Consultation on Incontinence [34].

Given the nature of the study, the response rate to the questionnaire and selection bias are inevitable. Participants could withdraw at any time and without reason, also potentially leading to bias. Furthermore, as data were collected via online surveys there could be a bias toward younger, more highly educated and/or less frail individuals who are more independent and tech-savvy for completing such a survey (vs. older, less educated and/or more frail individuals who are unable to complete an online survey). In addition, as target sample sizes were imposed for each subgroup, those who were eligible and responded fastest were included. It should also be noted that the questionnaires used (Kihon Checklist, OABSS and SF-12v2) have not been validated for online use. As this is a cross-sectional survey and analysis, any causality between frailty and OAB cannot be assessed. Also, any medication for OAB taken by respondents may affect their OABSS and HRQoL scores. Finally, the SF12v2 and SF-6D were used to assess HRQoL, and it is not known how sensitive these generic measures are at detecting any potential deterioration in HRQoL in the frail and OAB populations.

## Conclusions

These data highlight the statistically significant positive correlation between frailty and OAB among older adults in Japan and the higher prevalence of OAB among frail older adults, as well as of frailty among older adults with OAB. The study may also provide valuable data on the burden of OAB and frailty on older adults to healthcare professionals when considering future healthcare planning.

## Supplementary Information

**Additional file 1: Table S1.** Baseline OABSS, question responses and OAB severity.

**Additional file 2: Figure S1.** Adjusted means of HRQoL among frail participants with and without OAB.

## Data Availability

Researchers may request access to anonymized participant level data, trial level data and protocols from Astellas-sponsored clinical trials at www.clinicalstudydatarequest.com. For the Astellas criteria on data sharing see: https://clinicalstudydatarequest.com/Study-Sponsors/Study-Sponsors-Astellas.aspx.

## Abbreviations

ANOVA: Analysis of variance
BMI: Body mass index
CI: Confidence interval
HRQoL: Health-related quality of life
MCS: Mental Component Summary
MHLW: Ministry of Health, Labor and Welfare
NHWS: National Health and Wellness Survey
OAB: Overactive bladder
OABSS: Overactive bladder symptom score
PCS: Physical Component Summary
RCS: Role Component Summary
SD: Standard deviation
SF-12v2: Medical Outcomes Study 12-Item Short Form Survey Instrument version 2
