# Supplementary material for: A non-interventional cross-sectional re-contact study investigating the relationship between overactive bladder and frailty in older adults in Japan
Source: BMC Geriatr. 2022 Jan 21;22:68. doi: 10.1186/s12877-022-02756-7 (PMC8783467; doi:10.1186/s12877-022-02756-7)
Supplement: Supplementary file 1 — Additional file 1: Table S1. Baseline OABSS, question responses and OAB severity. [file 12877_2022_2756_MOESM1_ESM.docx]

**Additional file 1**

**Supplementary Table 1.** Baseline OABSS, question responses and OAB severity

| **Population 1** | **OAB (*n* = 566)** | | **Non-OAB (*n* = 2387)** | |
| --- | --- | --- | --- | --- |
|  | **Frail  (*n* = 150)** | **Non-frail  (*n* = 416)** | **Frail  (*n* = 287)** | **Non-frail  (*n* = 2100)** |
| Mean (SD) OABSS total | 6.21 (2.24) | 5.84 (2.00) | 2.28 (1.45) | 1.74 (1.34) |
| Mean (SD) OABSS Question 1 | 0.72 (0.59) | 0.74 (0.56) | 0.46 (0.53) | 0.43 (0.52) |
| Mean (SD) OABSS Question 2 | 1.50 (0.87) | 1.47 (0.90) | 1.13 (0.89) | 0.88 (0.82) |
| Mean (SD) OABSS Question 3 | 2.67 (0.87) | 2.53 (0.74) | 0.50 (0.54) | 0.30 (0.47) |
| Mean (SD) OABSS Question 4 | 1.32 (1.28) | 1.11 (1.09) | 0.19 (0.44) | 0.13 (0.35) |
| How many times do you typically urinate, from waking in the morning until sleeping at night? n (%) | | | | |
| ≤7  8–14  ≥15 | 53 (35.3)  86 (57.3)  11 (7.3) | 134 (32.2)  258 (62.0)  24 (5.8) | 160 (55.7)  123 (42.9)  4 (1.4) | 1214 (57.8)  865 (41.2)  21 (1.0) |
| How many times do you typically wake up to urinate, from sleeping at night until waking in the morning? n (%) | | | | |
| None  1  2  ≥3 | 14 (9.3)  71 (47.3)  41 (27.3)  24 (16.0) | 55 (13.2)  175 (42.1)  123 (29.6)  63 (15.1) | 70 (24.4)  137 (47.7)  53 (18.5)  27 (9.4) | 755 (36.0)  927 (44.1)  333 (15.9)  85 (4.0) |
| How often do you have a sudden desire to urinate, which is difficult to defer? n (%) | | | | |
| None  Less than once a week  Once a week or more  About once a week  2–4 times a day  ≥5 times a day | 0 (0.0)  0 (0.0)  83 (55.3)  41 (27.3)  19 (12.7)  7 (4.7) | 0 (0.0)  0 (0.0)  249 (59.9)  122 (29.3)  37 (8.9)  8 (1.9) | 150 (52.3)  131 (45.6)  6 (2.1)  0 (0.0)  0 (0.0)  0 (0.0) | 1466 (69.8)  628 (29.9)  6 (0.3)  0 (0.0)  0 (0.0)  0 (0.0) |
| How often do you leak urine because you cannot defer the sudden desire to urinate? n (%) | | | | |
| None  Less than once a week  Once a week or more  About once a week  2–4 times a day  ≥5 times a day | 54 (36.0)  29 (19.3)  44 (29.3)  15 (10.0)  4 (2.7)  4 (2.7) | 145 (34.9)  145 (34.9)  77 (18.5)  37 (8.9)  9 (2.2)  3 (0.7) | 237 (82.6)  46 (16.0)  3 (1.0)  1 (0.3)  0 (0.0)  0 (0.0) | 1848 (88.0)  241 (11.5)  11 (0.5)  0 (0.0)  0 (0.0)  0 (0.0) |
| OAB subgroup n (%) | | | | |
| Non-OAB  Mild OAB  Moderate OAB  Severe OAB | 0 (0.0)  66 (44.0)  80 (53.3)  4 (2.7) | 0 (0.0)  211 (50.7)  198 (47.6)  7 (1.7) | 287 (100.0)  0 (0.0)  0 (0.0)  0 (0.0) | 2100 (100.0)  0 (0.0)  0 (0.0)  0 (0.0) |

*Abbreviations: OAB* overactive bladder, *OABSS* overactive bladder symptom score
