# Supplementary material for: A non-interventional cross-sectional re-contact study investigating the relationship between overactive bladder and frailty in older adults in Japan
Source: BMC Geriatr. 2022 Jan 21;22:68. doi: 10.1186/s12877-022-02756-7 (PMC8783467; doi:10.1186/s12877-022-02756-7)

**Additional file 2**

**Supplementary Fig. 1** Adjusted means of HRQoL among frail participants with and without OAB. HRQoL, health-related quality of life; MCS, Mental Component Summary; OAB, overactive bladder; PCS, Physical Component Summary; RCS, Role Component Summary; SE, standard error; SF-12v2, Medical Outcomes Study 12-Item Short Form Survey Instrument version 2; SF-6D, 6-dimensional health state classification

Adjusted for age, sex, household income, living status, Charlson comorbidity index, body mass index, and exercise behavior; **p* < 0.05


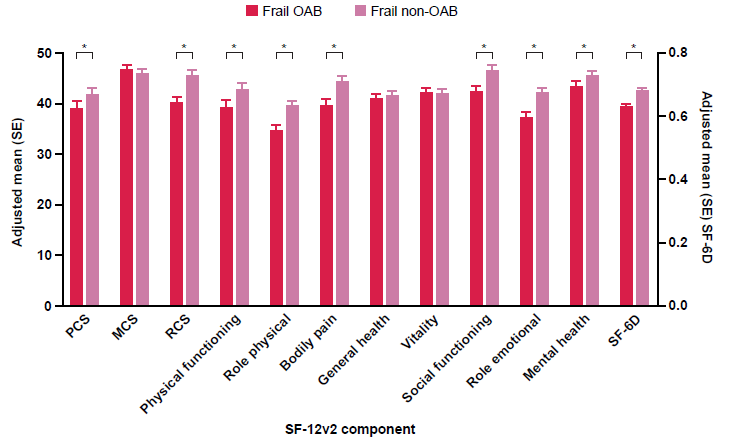

Supplement: Supplementary file 2 — Additional file 2: Figure S1. Adjusted means of HRQoL among frail participants with and without OAB. [file 12877_2022_2756_MOESM2_ESM.docx]
